# Prospective validation of the role of PET/CT in detecting disease after neoadjuvant chemotherapy in advanced ovarian cancer

**DOI:** 10.1007/s00330-024-10674-y

**Published:** 2024-03-09

**Authors:** Elaine Yuen Phin Lee, Philip Pun Ching Ip, Ka Yu Tse, Keith Wan Hang Chiu, Mandy Man Yee Chu, Yu Ka Chai, Philip Yuguang Wu, Jessica Yun Pui Law, Shuk Tak Kwok, Wan Kam Chiu, Hextan Yuen Sheung Ngan

**Affiliations:** 1 Department of Diagnostic Radiology, School of Clinical Medicine, LKS Faculty of Medicine, University of Hong Kong, Room 406, Block K, Queen Mary Hospital, 102 Pokfulam Road, Hong Kong, China; 2 Department of Pathology, School of Clinical Medicine, LKS Faculty of Medicine, University of Hong Kong, Hong Kong, China; 3 Department of Obstetrics and Gynaecology, School of Clinical Medicine, LKS Faculty of Medicine, University of Hong Kong, Hong Kong, China; 4 Department of Obstetrics and Gynaecology, United Christian Hospital, Hong Kong, China; 5 Department of Clinical Oncology, Pamela Youde Nethersole Eastern Hospital, Hong Kong, China; 6 Department of Obstetrics and Gynecology, Pamela Youde Nethersole Eastern Hospital, Hong Kong, China

**Keywords:** 2-[^18^F]FDG PET/CT, Contrast-enhanced CT, Ovarian cancer, Neoadjuvant chemotherapy, Chemotherapy response score

## Abstract

**Objectives:**

The study aimed to compare the diagnostic accuracies of 2-[^18^F]FDG PET/CT and contrast-enhanced CT (ceCT) after neoadjuvant chemotherapy (NACT) in advanced ovarian cancer (OC).

**Materials and methods:**

This study consisted historical observational cohort and prospective validation cohort. Patients with newly diagnosed stage III–IV OC scheduled for NACT were recruited, with imaging performed after three to six cycles of NACT before interval debulking surgery. Nineteen regions in the abdominopelvic cavity were scored for the presence and absence of disease, referenced to the intra-operative findings or histological specimens. Diagnostic metrics were compared using McNemar’s test.

**Results:**

In the historical cohort (23 patients, age 58 ± 13), 2-[^18^F]FDG PET had an overall accuracy (Acc) 82%, sensitivity (Sen) 38%, specificity (Spe) 97%, positive predictive value (PPV) 79% and negative predictive value (NPV) 82%; ceCT had an overall Acc 86%, Sen 64%, Spe 93%, PPV 75% and NPV 89%. In the prospective cohort (46 patients, age 59 ± 9), 2-[^18^F] FDG PET had an overall Acc 87%, Sen 48%, Spe 98%, PPV 84% and NPV 88%; ceCT had an overall Acc 89%, Sen 66%, Spe 95%, PPV 77% and NPV 91%. No significant difference was demonstrated between the two imaging modalities (*p* > 0.05). High false-negative rates were observed in the right subdiaphragmatic space, omentum, bowel mesentery and serosa. High omental metabolic uptake after NACT was associated with histological non-responders (*p* < 0.05).

**Conclusion:**

2-[^18^F]FDG PET/CT had no additional value over ceCT with comparable diagnostic accuracy in detecting disease after NACT in advanced OC.

**Clinical relevance statement:**

2-[^18^F]FDG PET/CT is not superior to contrast-enhanced CT in determining disease after neoadjuvant chemotherapy in advanced ovarian cancer; contrast-enhanced CT should be suffice for surgical planning before interval debulking surgery.

**Key Points:**

• *Additional value of 2-[*^18^*F]FDG PET/CT over contrast-enhanced CT is undefined in detecting disease after neoadjuvant chemotherapy.*

• *2-[*^18^*F]FDG PET/CT has comparable diagnostic accuracy compared to contrast-enhanced CT.*

• *Contrast-enhanced CT will be suffice for surgical planning after neoadjuvant chemotherapy.*

## Introduction

Over 60% of ovarian cancer (OC) present late at advanced-stage disease [1]. Disease prognosis depends on achieving complete cytoreduction at upfront primary debulking surgery (PDS) [2]. However, this is not achievable in a proportion of patients despite best effort. Neoadjuvant chemotherapy (NACT) followed by interval debulking surgery (IDS) is identified as a treatment alternative for patients who are likely to have residual disease after PDS or unfit for PDS. NACT/IDS was non-inferior to PDS in terms of survival rates [3, 4] and it reduced surgical invasiveness [5].

As NACT/IDS is increasingly adopted as treatment alternative for advanced OC [6], there is paucity of research to support how treatment response should be evaluated after NACT. Histopathological evaluation of tumour regression based on the NACT-induced tumour microenvironmental changes was prognostic and could be graded by the Chemotherapy Response Score (CRS) [7]. However, despite providing prognostic information in risk-stratifying patients for further therapies after IDS, the histopathological evaluation following NACT has little role in optimising selection of surgical candidates and surgical planning before IDS. The serum biomarker, CA-125, is widely studied in monitoring response to NACT [8], but exploratory abdominopelvic surgery like laparoscopy could transiently elevate the CA-125 level and could be problematic as a serum biomarker for monitoring response [9]. Novel serum biomarker, human epididymis protein 4 (HE4), has shown promising results as an adjunct to CA-125 [10], but not been widely adopted into clinical practice due to the relative high cost and limited availability.

Cross-sectional imaging offers treatment response assessment and surgical planning in OC undergoing NACT. Commonly used imaging modalities include contrast-enhanced computed tomography (ceCT), combined 2-deoxy-2-[^18^F]fluoro-d-glucose positron emission tomography/computed tomography (2-[^18^F]FDG PET/CT) and magnetic resonance imaging (MRI). Previous study had shown that ceCT had low negative predictive value in determining residual disease after NACT [11]. On the contrary, a predictive CT model that was initially developed in the PDS cohort was applicable to patients undergoing NACT and was predictive of optimal debulking at IDS [12]. Two studies based on 2-[^18^F]FDG PET/CT demonstrated its value in prediction of surgical outcome [13, 14].

There is a gap of knowledge in the literature in addressing the value of radiological assessment in patients with advanced OC undergoing NACT, especially the lack of head-to-head comparison between a widely available ceCT and advanced but more expensive 2-[^18^F]FDG PET/CT. There is a need to clarify the choice of imaging modality (between ceCT and 2-[^18^F]FDG PET/CT) in this cohort, so to make efficient use of the limited resources available and to reduce radiation burden. Furthermore, it is unclear if the metabolic response on 2-[^18^F]FDG PET/CT is associated with complete tumour regression on histology. Therefore, the aims of this study were to (1) examine the diagnostic efficacies of 2-[^18^F]FDG PET/CT and ceCT in determining disease following NACT in advanced OC, using historical observational cohort, followed by prospective validation, and (2) evaluate the relationship between metabolic uptake and histopathological response following NACT.

## Methodology

The study was approved by institutional review boards. Informed consents were obtained from all patients from the prospective cohort and waived in the retrospective cohort. The study adhered to the Declaration of Helsinki.

### Patients

The historical observational cohort was recruited between July 2008 and April 2019. The prospective validation cohort was consecutively recruited between June 2019 and July 2023. Both cohorts followed the same inclusion and exclusion criteria. The inclusion criteria were patients (1) with newly diagnosed and histologically proven FIGO stage III–IV OC; (2) who were medically fit for cytoreductive surgery with Eastern Cooperative Oncology Group (ECOG) performance status < 3; (3) who were unsuitable for PDS, either based on surgical evaluation (e.g. laparoscopy) or after imaging review and discussion at multi-disciplinary meeting according to ESGO 2017 recommendations [15]; (4) who would undergo NACT before IDS; (5) who had 2-[^18^F]FDG PET/CT paired with ceCT after NACT and before IDS; and (6) with histology known to be 2-[^18^F]FDG avid. Exclusion criterion was history of other malignancy, other than OC. Demographics and serial CA-125 were recorded for each recruit.

### Neoadjuvant chemotherapy/interval debulking surgery

Standard NACT regimen included three to six cycles of platinum and taxane-based chemotherapy (carboplatin AUC5 and paclitaxel 175 mg/m^2^ body surface area, administered at interval of 3 weeks) before IDS. All IDS were performed by board-certified gynae-oncologists with more than 10 years of post-fellowship experiences and assisted by fellows. All IDS were performed with intention of complete cytoreduction. Standard surgical procedure consisted of resection of pelvic tumours, omentum, pelvic and paraaortic lymph nodes, and extra-pelvic tumours. Additional extensive surgical procedures were defined as procedures involving diaphragmatic peritonectomy, resection of porta hepatis disease, hepatectomy, splenectomy or pancreatectomy, cystectomy and bowel resection. The immediate surgical outcome from IDS was determined through visual inspection of the abdominopelvic cavity at the end of surgery. Complete IDS was defined as no macroscopic residual disease at the end of surgery; the presence of residual disease regardless of size would be considered incomplete IDS. Biopsy would be taken in regions without macroscopic disease for systematic documentation whenever feasible and safe. For unresectable disease, similar biopsy will be taken for reference. Histological assessment on the resected specimens at IDS were evaluated by board-certified pathologist specialised in gynaecological pathology (over 15 years post-fellowship) and reviewed at the weekly multi-disciplinary meeting. Histopathological specimens were taken as gold standard. In the event where resection was not performed and biopsy could not be safely taken (e.g., miliary serosal disease), the intra-operative surgical findings were taken as standard of reference.

### Chemotherapy response score

Same board-certified pathologist examined all omental specimens and graded the response to NACT based on CRS [7]. In short, CRS 1 referred to omental specimen with mainly viable tumour and minimal regression-associated fibro-inflammatory changes; CRS 2 was reserved for specimen with multifocal or diffuse fibro-inflammatory changes, but residual viable tumour was easily identifiable; and CRS 3 represented mainly regression with few irregularly scattered individual tumour cells or no residual tumour identified. CRS 1/2 was defined as histological non-responder, while CRS 3 was considered histological responder.

### Imaging acquisitions

2-[^18^F]FDG PET/CT and ceCT were acquired after three to six cycles of NACT before scheduled IDS. Patients were fasted for 6 h prior to 2-[^18^F]FDG PET/CT examination with glucose level below 144 mg/dl at the time of 2-[^18^F]FDG injection (weight-based: weight (kg) × 0.13 mCi) using dedicated PET/CT scanner (Discovery 610; 64-slice, GE Healthcare Bio-Sciences Corp.) 60 min following 2-[^18^F]FDG injection, covering the skull base to the upper thighs. To reduce radiation exposure, ceCT was performed as part of the 2-[^18^F]FDG PET/CT on the same visit with the following parameters: field of view; 50 cm; 120 kVp; 200–400 mA; 0.5 s/CT rotation, pitch 0.984:1 with injection of intravenous contrast medium (1.5 mL/kg) at a rate of 2.0 mL/s, and acquired at 70 s following intravenous contrast injection in the porto-venous phase based on the same body coverage. This would be subsequently used for attenuation correction and PET images will be reconstructed using an ordered-subset expectation maximisation iterative algorithm (14 subsets and two iterations).

### Image analysis

Two experienced board-certified radiologists (R1, over 10 years post-fellowship and R2, over 5 years post-fellowship, with additional training in interpretation of 2-[^18^F]FDG PET/CT in an university-based high-volume PET/CT centre) reviewed the 2-[^18^F]FDG PET/CT and ceCT in separate reading sessions. There was at least 4 weeks’ interval between the 2-[^18^F]FDG PET/CT and ceCT reading sessions; both radiologists were blinded to the surgical and histological findings. R1 re-evaluated the two set of images at least 4 weeks apart from the initial read to evaluate the intra-observer consistency. The abdominopelvic cavity was divided into the following anatomical regions to allow systematic scoring: subdiaphragmatic surfaces, liver serosa, gastric serosa, splenic hilum, paracolic gutters, omentum, mesentery and serosa of small and large bowels, pelvic and paraaortic nodal chains, and central pelvis. These regions were individually scored. Discrepancy between the two radiologists was resolved in consensus. Baseline imaging (including other modalities) before NACT was referred for interpretation.

On 2-[^18^F]FDG PET/CT, any distinct 2-[^18^F]FDG uptake more than background physiologic uptake in the abdomen and pelvis would be considered positive and was evaluated by 5-point scale (1, no FDG uptake; 2, FDG uptake below the mediastinal blood pool; 3, FDG uptake above the mediastinal blood pool but below the liver background; 4, FDG uptake above the liver background; 5, FDG uptake substantially above liver background or new abnormal uptake). On ceCT, abnormal soft tissue of any size or abnormal enhancement was considered positive (1, no disease; 2, benign; 3, indeterminate; 4, probable malignant; 5, definite malignant or new lesion). Scores 3–5 were considered positive for presence of disease.

The maximum standardised uptake value (SUVmax) of the omental disease was recorded. In case where the omental disease had completely resolved, this would be arbitrarily labelled as “0” for the purpose of data analysis. In addition, the ratio between the omental SUVmax and background liver SUVmax was computed.

### Statistical analysis

Both parametric and non-parametric tests were used to compare the demographic and clinical differences between the two cohorts depending on the data distribution. Categorical comparison was made with chi-square test. Inter-observer and intra-observer variability were evaluated by intraclass correlation coefficient (ICC). Region-based analysis was performed and the diagnostic characteristics of 2-[^18^F]FDG PET/CT and ceCT were described by accuracy (Acc), sensitivity (Sen), specificity (Spe), positive predictive value (PPV) and negative predictive value (NPV). The diagnostic characteristics of the two imaging modalities were compared by McNemar’s test. The three regions with the highest false-negative rates were identified. Mann–Whitney *U* test was used to determine the differences in omental SUVmax or ratio (omental/liver SUVmax) amongst the three-tier CRS. We employed SciPy library and the statsmodels library in Python (version 3.9.16) IDE for all the statistical analyses. The significance level was set at 0.05.

## Results

### Patients

**Fig. 1:**
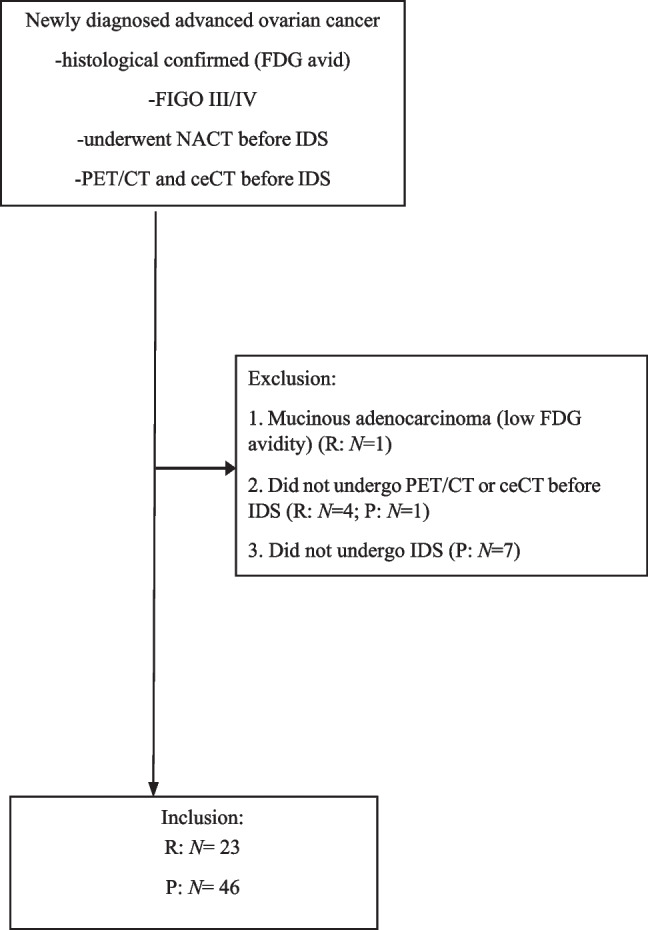
Flow diagram of patient inclusion and exclusion criteria. *R* historical retrospective cohort, *P* prospective cohort

The historical observational cohort consisted of 23 patients and the prospective validation cohort was made up of 46 patients (Fig. 1). The demographics and clinical details of both cohorts are tabulated in Table 1. Marginal difference was observed in the distribution of the FIGO stages between the two cohorts with more FIGO stage IV disease in the historical cohort. We observed difference in the number of NACT cycles administered between the two cohorts with a median of four cycles in the historical cohort and three cycles in the prospective cohort. This was thought to be due to the shift in clinical practice over the years, in that IDS was performed earlier now than the historical cohort. However, no difference was observed in the rate of achieving complete cytoreduction after IDS in the two cohorts. There was no correlation between the percentage change in CA-125 and the presence of residual disease after IDS.

**Table 1:** Demographics and clinical information between historical observational retrospective cohort and prospective cohort. Age was reported in mean ± standard deviation. CA-125 was presented in median and range

Cohorts	Historical observational	Prospective validation	*p* values
*N*	23	46	
Age (years)	58 ± 13	59 ± 9	0.670
FIGO			0.042
III	6	28	
IV	17	18	
No. cycles of NACT			< 0.005
3	7	35	
4–6	16	11	
CA-125			
Baseline	2454 (195–40,740)	967 (96–35,119)	0.014
Pre-IDS	45 (8–6576)	88 (6–982)	0.367
% change	98 (39–100)	95 (− 21–100)	0.011
Time between imaging and IDS (days)	15 (10–38)	14 (6–46)	0.125
Residual disease after IDS			0.722
No	19	35	
Yes	4	11	
Histology			0.072
HGSC	18	44	
Non-HGSC	5	2	

*HGSC* high-grade serous adenocarcinoma, *IDS* interval debulking surgery, *NACT* neoadjuvant chemotherapy

### Diagnostic characteristics

All patients underwent either ceCT or 2-[^18^F]FDG PET/CT as baseline imaging, except for one patient in the prospective cohort who had baseline MRI, which was subsequently evaluated by diagnostic laparoscopy to confirm the MRI findings (historical cohort: ceCT *n* = 3, 2-[^18^F]FDG PET/CT *n* = 20; prospective cohort: ceCT *n* = 9, 2-[^18^F]FDG PET/CT *n* = 36 and MRI *n* = 1). The inter-observer ICCs for both cohorts were 0.893 for PET/CT and 0.697 for ceCT, which were considered as excellent and good, respectively, while the intra-observer ICCs were 0.997 for PET/CT and 0.946 for ceCT, both considered excellent [16].

The diagnostic characteristics of 2-[^18^F]FDG PET/CT and ceCT from the two cohorts are tabulated in Tables 2 and 3. In the prospective cohort, 2 patients had severe adhesions in the upper abdomen, rendering 5 regions inaccessible for surgical evaluation, namely right subdiaphragmatic region in 2 patients, left subdiaphragmatic, perihepatic/subhepatic and liver serosa/hepatic hilum in 1 patient, respectively. These regions were excluded from further analysis due to lack of standard of reference.

**Table 2:** Historical observational retrospective cohort. The diagnostic metrics of (A) PET/CT and (B) ceCT in detecting disease after NACT with histology or intra-operative evaluation at IDS as standard of reference. *Acc* accuracy, *ceCT* contrast-enhanced CT, *FN* false negative, *FP* false positive, *IDS* interval debulking surgery, *LN* lymph node metastasis, *NACT* neoadjuvant chemotherapy, *NPV* negative predictive value, *PPV* positive predictive value, *Sen* sensitivity, *Spe* specificity, *TN* true negative, *TP* true positive

A	Regions	TP	FP	TN	FN	Acc	Sen	Spe	PPV	NPV
PET/CT	R Subdiaphragmatic	0	0	13	10	0.57	0.00	1.00	–	0.57
	L Subdiaphragmatic	1	2	16	4	0.74	0.20	0.89	0.33	0.80
	Perihepatic/subhepatic	0	0	18	5	0.78	0.00	1.00	–	0.78
	Liver serosa or hilum/porta hepatis	1	0	19	3	0.87	0.25	1.00	1.00	0.86
	Gastric serosa	0	0	21	2	0.91	0.00	1.00	–	0.91
	Pancreas/lesser sac	0	0	22	1	0.96	0.00	1.00	–	0.96
	Splenic serosa	0	0	20	3	0.87	0.00	1.00	–	0.87
	Splenic hilum	0	0	17	6	0.74	0.00	1.00	–	0.74
	R paracolic gutter	0	1	20	2	0.87	0.00	0.95	0.00	0.91
	L paracolic gutter	1	3	18	1	0.83	0.50	0.86	0.25	0.95
	Omentum	8	1	4	10	0.52	0.44	0.80	0.89	0.29
	Small bowel mesentery/serosa	0	0	16	7	0.70	0.00	1.00	–	0.70
	Large bowel mesentery/serosa	4	2	11	6	0.65	0.40	0.85	0.67	0.65
	R paraarotic LN	2	0	20	1	0.96	0.67	1.00	1.00	0.95
	L paraaortic LN	2	0	21	0	1.00	1.00	1.00	1.00	1.00
	Aortocaval LN	2	0	21	0	1.00	1.00	1.00	1.00	1.00
	R pelvic LN	1	1	20	1	0.91	0.50	0.95	0.50	0.95
	L pelvic LN	3	0	19	1	0.96	0.75	1.00	1.00	0.95
	Central pelvis	16	1	1	5	0.74	0.76	0.50	0.94	0.17
	Overall	**41**	**11**	**317**	**68**	**0.82**	**0.38**	**0.97**	**0.79**	**0.82**
B	Regions	TP	FP	TN	FN	Acc	Sen	Spe	PPV	NPV
ceCT	R Subdiaphragmatic	4	2	13	4	0.74	0.50	0.87	0.67	0.76
	L Subdiaphragmatic	4	5	14	0	0.78	1.00	0.74	0.44	1.00
	Perihepatic/subhepatic	1	2	17	3	0.78	0.25	0.89	0.33	0.85
	Liver serosa or hilum/porta hepatis	1	0	19	3	0.87	0.25	1.00	1.00	0.86
	Gastric serosa	0	0	21	2	0.91	0.00	1.00	–	0.91
	Pancreas/lesser sac	0	1	21	1	0.91	0.00	0.95	0.00	0.95
	Splenic serosa	1	1	19	2	0.87	0.33	0.95	0.50	0.90
	Splenic hilum	4	1	16	2	0.87	0.67	0.94	0.80	0.89
	R paracolic gutter	1	2	19	1	0.87	0.50	0.90	0.33	0.95
	L paracolic gutter	2	2	19	0	0.91	1.00	0.90	0.50	1.00
	Omentum	12	1	4	6	0.70	0.67	0.80	0.92	0.40
	Small bowel mesentery/serosa	2	0	16	5	0.78	0.29	1.00	1.00	0.76
	Large bowel mesentery/serosa	6	2	11	4	0.74	0.60	0.85	0.75	0.73
	R paraarotic LN	2	1	19	1	0.91	0.67	0.95	0.67	0.95
	L paraaortic LN	2	0	21	0	1.00	1.00	1.00	1.00	1.00
	Aortocaval LN	2	0	21	0	1.00	1.00	1.00	1.00	1.00
	R pelvic LN	1	1	20	1	0.91	0.50	0.95	0.50	0.95
	L pelvic LN	3	0	19	1	0.96	0.75	1.00	1.00	0.95
	Central pelvis	19	1	1	2	0.87	0.90	0.50	0.95	0.33
	Overall	**67**	**22**	**310**	**38**	**0.86**	**0.64**	**0.93**	**0.75**	**0.89**

**Table 3:** Prospective validation cohort. The diagnostic metrics of (A) PET/CT and (B) ceCT in detecting disease after NACT with histology or intra-operative evaluation at IDS as standard of reference. *Acc* accuracy, *ceCT* contrast-enhanced CT, *FN* false negative, *FP* false positive, *IDS* interval debulking surgery, *LN* lymph node metastasis, *NACT* neoadjuvant chemotherapy, *NPV* negative predictive value, *PPV* positive predictive value, *Sen* sensitivity, *Spe* specificity, *TN* true negative, *TP* true positive

A	Regions		TP	FP	TN	FN	Acc	Sen	Spe	PPV	NPV
PET/CT	R Subdiaphragmatic		6	0	28	10	0.77	0.38	1.00	1.00	0.74
	L Subdiaphragmatic		3	1	39	2	0.93	0.60	0.98	0.75	0.95
	Perihepatic/subhepatic		2	3	38	2	0.89	0.50	0.93	0.40	0.95
	Liver serosa or hilum/porta hepatis		3	0	40	2	0.96	0.60	1.00	1.00	0.95
	Gastric serosa		0	0	46	0	1.00	–	1.00	–	1.00
	Pancreas/lesser sac		0	0	44	2	0.96	0.00	1.00	–	0.96
	Splenic serosa		0	0	44	2	0.96	0.00	1.00	–	0.96
	Splenic hilum		0	0	44	2	0.96	0.00	1.00	–	0.96
	R paracolic gutter		5	3	31	7	0.78	0.42	0.91	0.63	0.82
	L paracolic gutter		4	1	32	9	0.78	0.31	0.97	0.80	0.78
	Omentum		20	0	11	15	0.67	0.57	1.00	1.00	0.42
	Small bowel mesentery/serosa		4	2	29	11	0.72	0.27	0.94	0.67	0.73
	Large bowel mesentery/serosa		9	1	21	15	0.65	0.38	0.95	0.90	0.58
	R paraarotic LN		0	2	43	1	0.93	0.00	0.96	0.00	0.98
	L paraaortic LN		0	1	44	1	0.96	0.00	0.98	0.00	0.98
	Aortocaval LN		0	2	44	0	0.96	–	0.96	0.00	1.00
	R pelvic LN		0	0	44	2	0.96	0.00	1.00	–	0.96
	L pelvic LN		0	1	43	2	0.93	0.00	0.98	0.00	0.96
	Central pelvis		31	0	7	8	0.83	0.79	1.00	1.00	0.47
	Overall		**87**	**17**	**672**	**93**	**0.87**	**0.48**	**0.98**	**0.84**	**0.88**
B	Regions	TP		FP	TN	FN	Acc	Sen	Spe	PPV	NPV
ceCT	R Subdiaphragmatic	9		0	28	7	0.84	0.56	1.00	1.00	0.80
	L Subdiaphragmatic	2		5	35	3	0.82	0.40	0.88	0.29	0.92
	Perihepatic/subhepatic	3		7	34	1	0.82	0.75	0.83	0.30	0.97
	Liver serosa or hilum/porta hepatis	3		1	39	2	0.93	0.60	0.98	0.75	0.95
	Gastric serosa	0		1	45	0	0.98	–	0.98	0.00	1.00
	Pancreas/lesser sac	0		0	44	2	0.96	0.00	1.00	–	0.96
	Splenic serosa	1		0	44	1	0.98	0.50	1.00	1.00	0.98
	Splenic hilum	0		0	44	2	0.96	0.00	1.00	–	0.96
	R paracolic gutter	7		6	28	5	0.76	0.58	0.82	0.54	0.85
	L paracolic gutter	8		4	29	5	0.80	0.62	0.88	0.67	0.85
	Omentum	29		0	11	6	0.87	0.83	1.00	1.00	0.65
	Small bowel mesentery/serosa	6		2	29	9	0.76	0.40	0.94	0.75	0.76
	Large bowel mesentery/serosa	13		4	18	11	0.67	0.54	0.82	0.76	0.62
	R paraarotic LN	0		0	45	1	0.98	0.00	1.00	–	0.98
	L paraaortic LN	0		1	44	1	0.96	0.00	0.98	0.00	0.98
	Aortocaval LN	0		0	46	0	1.00	–	1.00	–	1.00
	R pelvic LN	1		1	43	1	0.96	0.50	0.98	0.50	0.98
	L pelvic LN	0		2	42	2	0.91	0.00	0.95	0.00	0.95
	Central pelvis	36		2	5	3	0.89	0.92	0.71	0.95	0.63
	Overall	**118**		**36**	**653**	**62**	**0.89**	**0.66**	**0.95**	**0.77**	**0.91**

In the historical cohort, overall low sensitivity was observed in both modalities, 2-[^18^F]FDG PET/CT 38% and ceCT 64%. High false-negative rates, ranging from 26 to 43%, were found in the following regions on 2-[^18^F]FDG PET/CT, namely the right subdiaphragmatic region (10/23), omentum (10/23) and mesenteric and serosal disease of both the small (7/23) and large bowel (6/23). Similar regions were missed on ceCT but fewer, with false-negative rates of 17–26%: right subdiaphragmatic region (4/23), omentum (6/23) and mesenteric and serosal disease of the small bowel (5/23) and the large bowel (4/23).

**Fig. 2:**
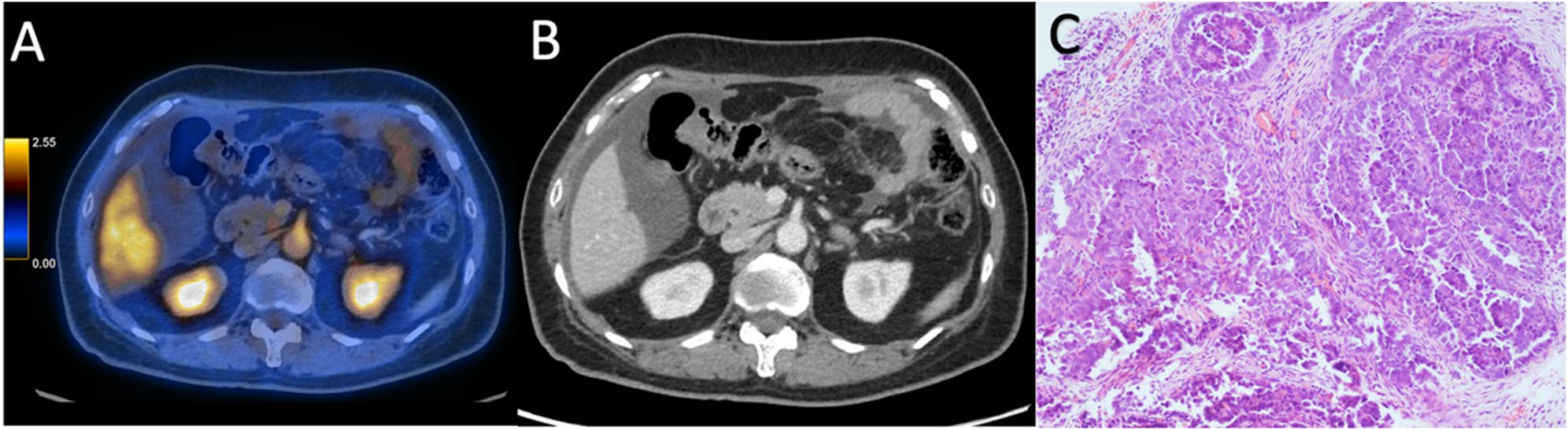
A 63-year-old patient with FIGO IIIC high-grade serous adenocarcinoma of the ovary underwent three cycles of NACT. **A** Axial fused FDG PET/CT showed no uptake in the omentum. **B** Axial contrast-enhanced CT showed fat stranding in the omentum. **C** Omental specimen (haematoxylin and eosin stain × 10): viable tumour cells with little evidence of therapy response, compatible with CRS 1. CRS, chemotherapy response score; NACT, neoadjuvant chemotherapy

**Fig. 3:**
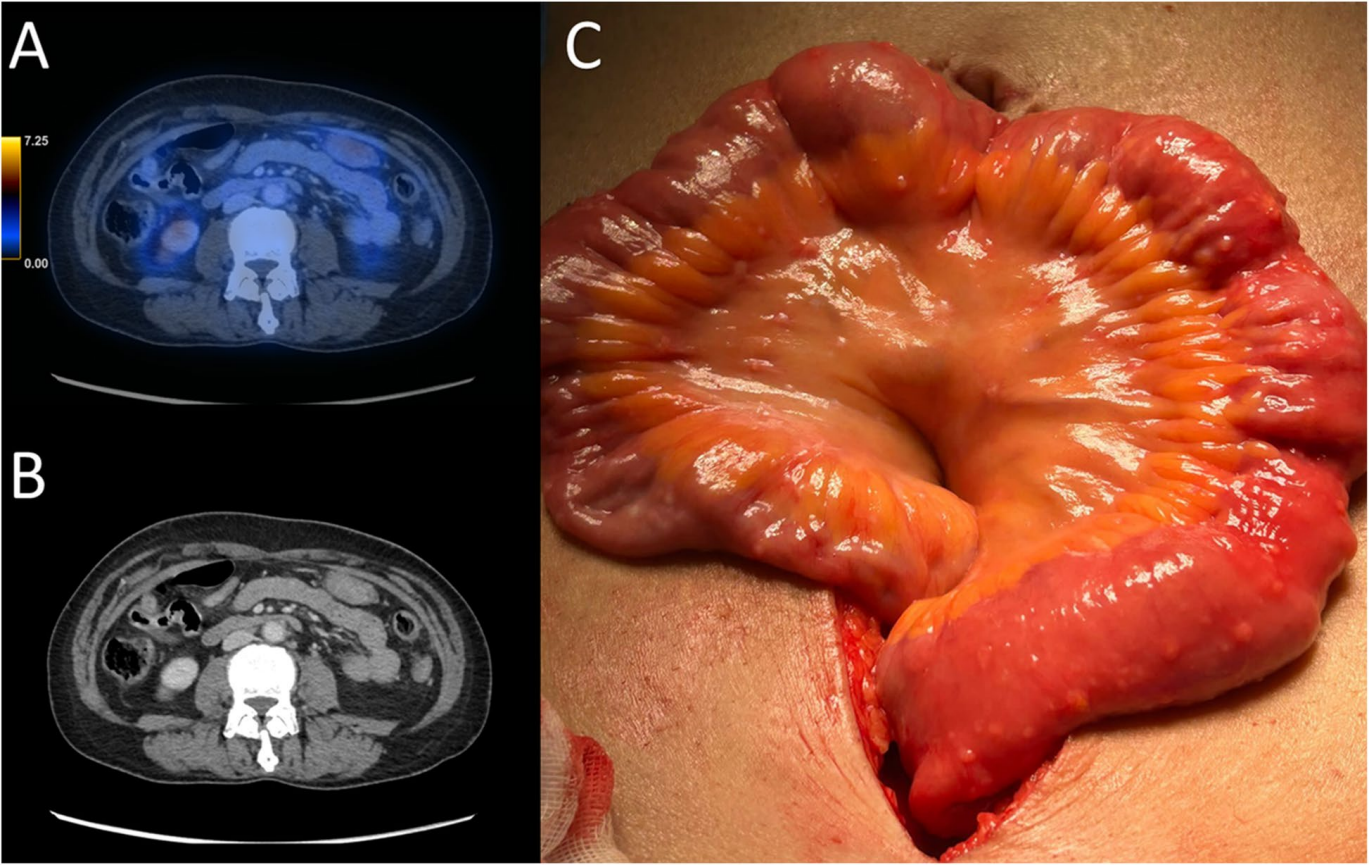
A 52-year-old patient with FIGO IV high-grade serous adenocarcinoma of the ovary underwent five cycles of NACT. **A**, **B** Axial fused FDG PET/CT and contrast-enhanced CT showed complete resolution of the peritoneal disease, including the small bowel serosal disease with no abnormal bowel uptake nor nodularity, bowel dilatation or bowel wall thickening could be observed. **C** Intra-operative finding at IDS showed diffused small bowel serosal deposits as tiny nodules on the surface of the small bowel, resulting in incomplete cytoereduction. IDS, interval debulking surgery; NACT, neoadjuvant chemotherapy

The findings were validated in the prospective cohort, the overall sensitivity remained low for 2-[^18^F]FDG PET/CT at 48% and ceCT at 66%. The three regions with the highest false-negative rates on 2-[^18^F]FDG PET/CT were observed in the right subdiaphragmatic region (10/44), omentum (15/46) (Fig. 2) and mesenteric and serosal disease of both the small (11/46) (Fig. 3) and large bowel (15/46). On ceCT, high false-negative rates were identified in the right subdiaphragmatic region (7/44), mesenteric and serosal disease of both the small (9/46) and large bowel (11/46). Omental disease was missed on ceCT but less frequent than the historical cohort (6/46).

### Metabolic uptake and histopathological response

**Fig. 4:**
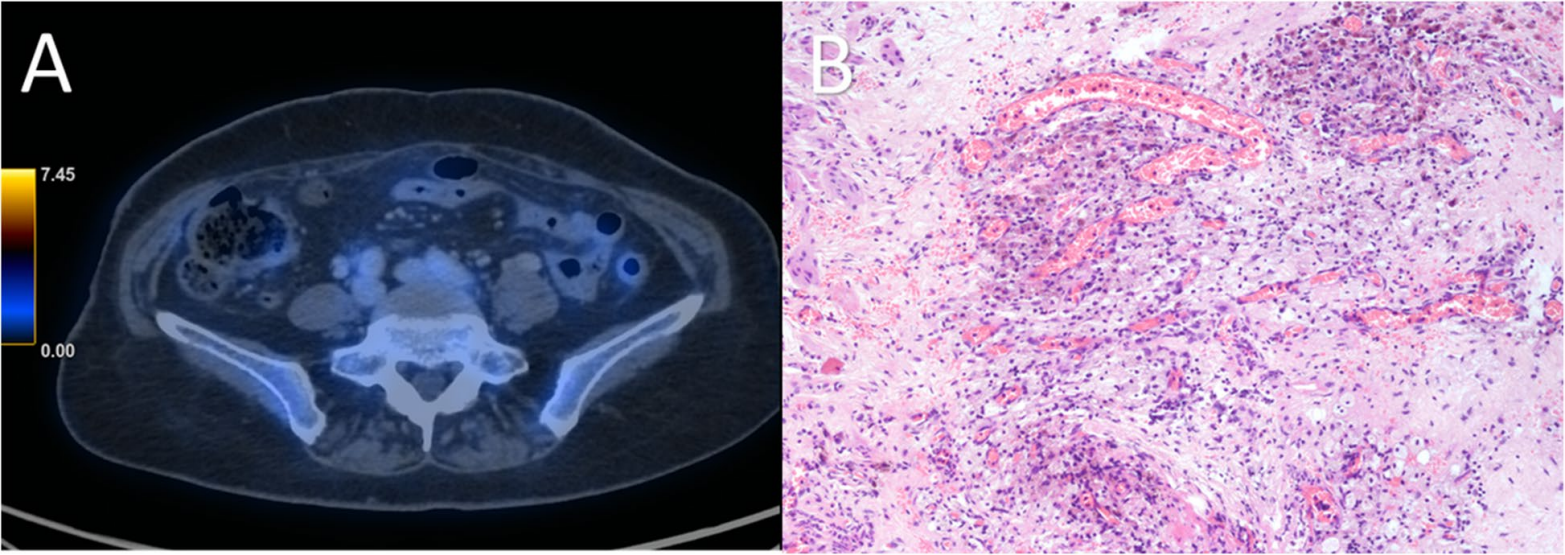
A 66-year-old patient with FIGO IV high-grade serous adenocarcinoma of the ovary underwent three cycles of NACT. **A** Axial fused FDG PET/CT showed no uptake in the omentum. **B** Omental specimen (haematoxylin and eosin stain × 10) with vascular congestion, foamy and haemosiderin-laden macrophages but no viable tumour cells, compatible with CRS 3. CRS, chemotherapy response score; NACT, neoadjuvant chemotherapy

**Fig. 5:**
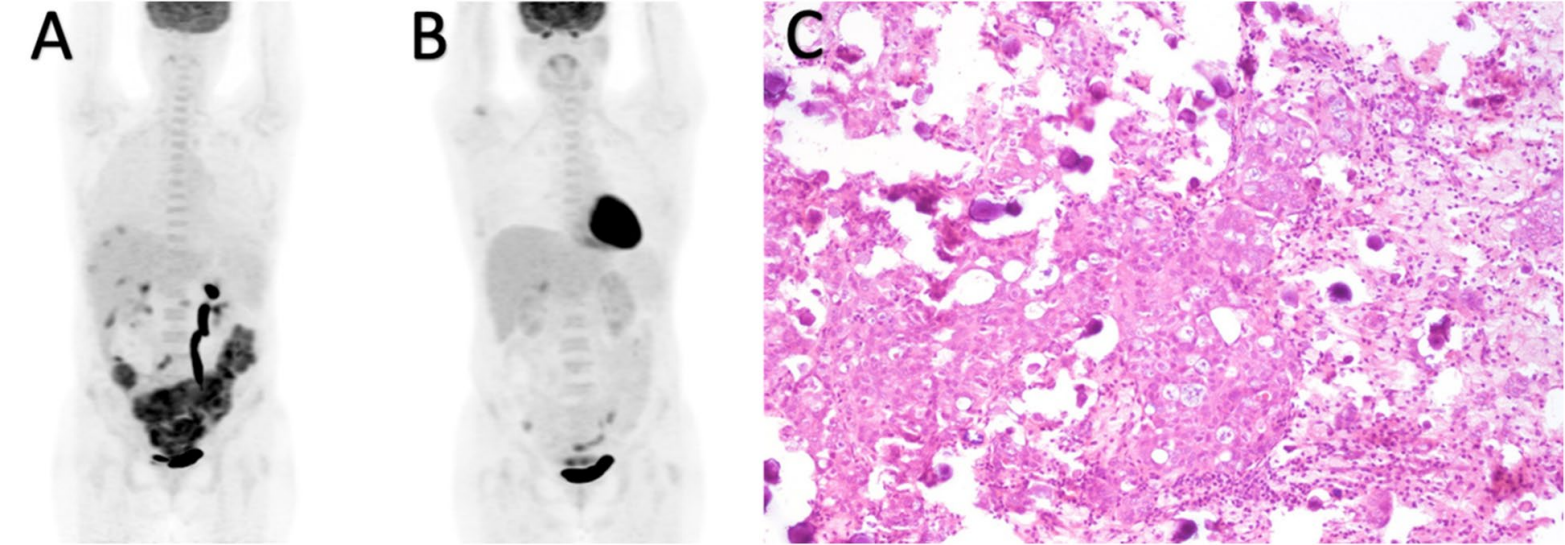
A 55-year-old patient with FIGO IIIC high-grade serous adenocarcinoma of the ovary underwent three cycles of NACT. **A** Maximum intensity project FDG PET before treatment showed extensive peritoneal disease spread with omental “cake”. **B** Following three cycles of NACT, there was good partial response but residual uptake remained; omental SUVmax 2.8, ratio 1.4. **C** Omental specimen (haematoxylin and eosin stain × 4) showed an aggregate of viable tumour cells surrounded by macrophages and scattered psammoma bodies signifying therapy response, compatible with CRS 2. CRS, chemotherapy response score

**Fig. 6:**
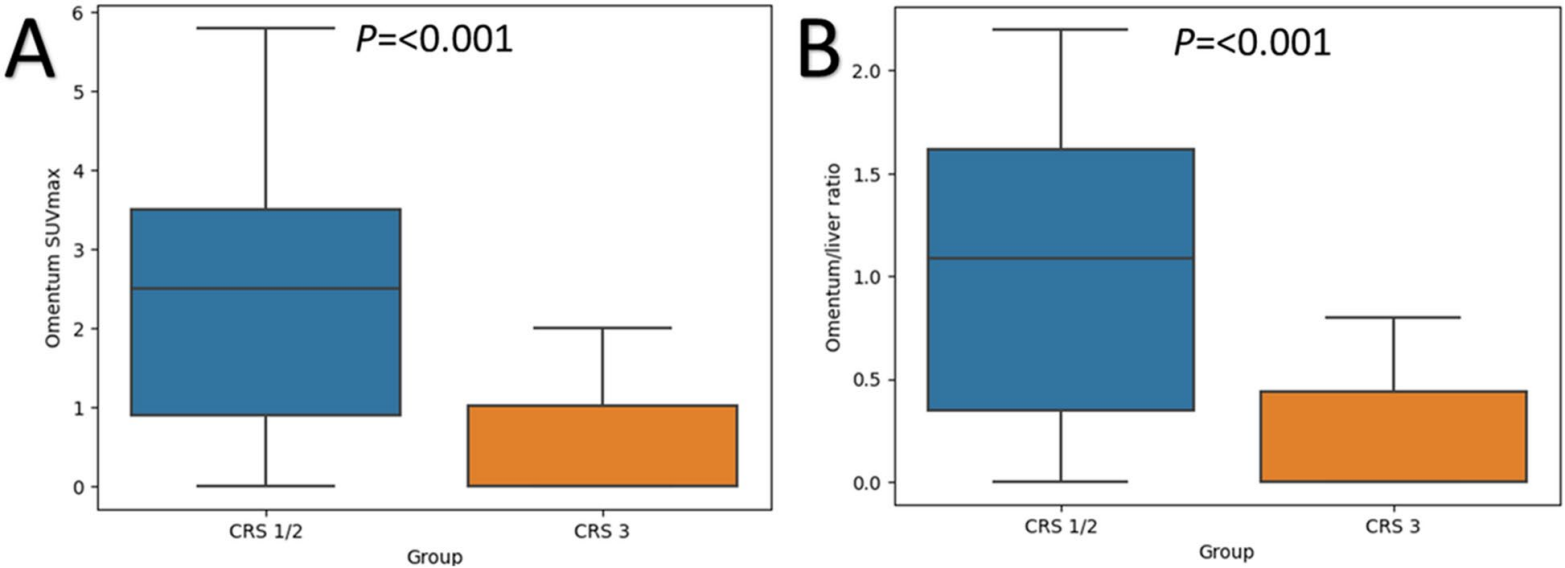
Box-plots showing the differences in (**A**) omental SUVmax and (**B**) SUVmax ratio between CRS 1/2 and CRS 3, histological non-responders and responders, respectively

Amongst the 46 patients in the prospective cohort, there was no omental tissue resected for one patient based on negative intra-operative survey, and therefore was excluded from the analysis. We demonstrated significant differences in the metabolic uptake of the omental disease and histopathological responses, CRS 1/2 (median SUVmax 2.1, SUVmax ratio 0.9) and CRS 3 (median SUVmax 0, SUVmax ratio 0). There was a negative relationship between omental SUVmax or SUVmax ratio and histopathological response, and higher metabolic uptake was associated with CRS 1/2, both *p* < 0.05 (Figs. 4, 5 and 6).

## Discussion

In this study, we found that 2-[^18^F]FDG PET/CT had comparable diagnostic metrics compared to ceCT in patients with advanced OC who underwent NACT, first observed in the historical cohort and subsequently validated prospectively. Both modalities suffered from low sensitivity with high false-negative findings in the right subdiaphragmatic space, omentum, bowel mesentery and serosa. Higher omental uptake was associated less favourable histopathological response (CRS 1/2) after NACT.

After completion of NACT, ceCT is commonly arranged to evaluate chemotherapy response and at the same time provide surgical planning and roadmap for gynae-oncologists. Early work showed that sequential PET was able to predict response to NACT in advanced OC and the metabolic response was more prognostic of survival outcome than biochemical (CA-125), clinical and histopathologic responses [17]. Subsequent 2-[^18^F]FDG PET/CT studies confirmed the prognostic value of metabolic response in predicting disease outcome, in that metabolic responders were more likely to have longer progression-free survival, overall survival or both [13, 14, 18, 19].

However, the role of 2-[^18^F]FDG PET/CT in surgical planning after NACT is less well-defined and lack head-to-head comparison with more widely available and less costly ceCT. In our study, we did not find superiority of 2-[^18^F]FDG PET/CT in region-based analysis compared to ceCT. Both modalities suffered from low sensitivity due to high false-negative rates in certain regions of the abdominopelvic cavity, namely the right subdiaphragmatic space, omentum, bowel mesentery and serosa. This was likely because of the reduction in tumour burden following NACT, rendering the disease to miliary or non-measurable thickening, and difficult to detect on both modalities. The improvement or resolution of ascites, which could act as a negative contrast on ceCT, would make small volume of peritoneal disease less conspicuous. Residual uptake on 2-[^18^F]FDG PET/CT could be masked by adjacent physiological activity, for example background liver uptake in the right subdiaphragmatic region and diffuse bowel activity in the vicinity of the bowel mesentery and serosa. Both 2-[^18^F]FDG PET/CT and ceCT missed omental disease with small disease foci or microscopic disease below the resolution of these imaging modalities following treatment response to NACT. Clinically, this would have less impact on surgical planning as the standard IDS would include omentectomy to remove microscopic disease.

Hynninen et al previously reported both 2-[^18^F]FDG PET/CT and ceCT performed before PDS or NACT had poor sensitivity in detecting disease in the right upper quadrant and small bowel mesentery, concordant to our findings. The study showed no difference in the number of patients requiring extensive upper abdominal surgical procedures with either modality [20]. Various 2-[^18^F]FDG PET/CT features were identified as predictors of incomplete cytoreduction, including hypermetabolic large bowel mesenteric implant, metabolic metrics, number of 2-[^18^F]FDG avid peritoneal sites and modified peritoneal cancer index score in mixed cohorts of patients, from primary to recurrent OC [21–25].

Risum et al identified large bowel mesenteric implant on 2-[^18^F]FDG PET/CT as an independent predictor of incomplete cytoreduction [21]. Subsequently, in a larger cohort consisted of 343 FIGO stage III/IV OC who underwent PDS, Shim et al identified 5 2-[^18^F]FDG PET/CT features as significant predictors of incomplete cytoreduction to be included in the nomogram, namely diaphragmatic disease, presence of ascites, peritoneal carcinomatosis, small bowel mesenteric implant and tumoural uptake ratio [26]. These studies highlighted the impact of mesenteric implants on complete cytoreduction, and our study showed that both modalities were not sufficiently sensitive in detecting mesenteric disease after NACT. Therefore, careful intra-operative evaluation at IDS remains important.

We demonstrated that higher metabolic uptake or SUVmax ratio was associated with unfavourable histopathological response, CRS 1/2. Our results were concordant with others showing an association between metabolic and histopathological responses, despite differences in the timing of 2-[^18^F]FDG PET/CT and methods of analysis [14, 27–29]. The metabolic change after three to four cycles of NACT could identify histopathological non-responder and a substantial decrease in SUVmax was required to achieve favourable histopathological response [27]. Using various thresholds, ranging from 40 to 100% reduction in SUVmax, they could predict histopathological responders [14, 27–29]. Earlier studies also used different histopathological scoring system [27–29], whereas CRS used in our study is a validated three-tier score that has been shown to be reproducible amongst pathologists and recommended by International Collaboration on Cancer Reporting to be included in standardised reporting [7, 30]. Furthermore, we used the liver as internal reference to derive the SUVmax ratio without relying on the baseline 2-[^18^F]FDG PET/CT, which could be unavailable in many cases. Despite the negative relationship, significant overlap was observed in the metabolic uptake between CRS 1/2 and CRS 3, especially when CRS 1/2 could be associated with complete resolution of metabolic uptake on 2-[^18^F]FDG PET/CT with undetectable microscopic disease.

The study suffered from several limitations. First, the numbers of patients were small for both cohorts, which could introduce bias in the data analysis but the prospective validation on the retrospective observation was a strength of the study. Second, the incidence of disease involvement was low in certain anatomical regions, for example gastric serosa, which could inflate the regional accuracy. Third, as not all patients had baseline 2-[^18^F]FDG PET/CT, pre-post evaluation based on PET Response Criteria in Solid Tumors (PERCIST) could not be performed. Fourth, histopathological confirmation was not possible for all regions and intra-operative assessment was used as surrogate with its inherent limitations in that adhesions and NACT-induced changes in the abdominopelvic cavity could affect the intra-operative evaluation at IDS [31]. Fifth, we only explored the metabolic uptake with CRS in omental samples but not adnexal specimens. However, CRS was most reproducible in omentum and less reproducible in adnexa [7].

## Conclusions

In conclusion, 2-[^18^F]FDG PET/CT had comparable diagnostic accuracy in region-based analysis when compared with ceCT; both suffered from low sensitivity, especially in determining disease in the right subdiaphragmatic region, omentum, bowel mesentery and serosa. The SUVmax and SUVmax ratio were associated with CRS, and higher metabolic uptake was observed in histopathological non-responders.

## Abbreviations

2-[^18^F]FDG: 2-Deoxy-2-[^18^F]fluoro-d-glucose
Acc: Accuracy
ceCT: Contrast-enhanced CT
CRS: Chemotherapy response score
HE4: Human epididymis protein 4
ICC: Intraclass correlation coefficient
IDS: Interval debulking surgery
MRI: Magnetic resonance imaging
NACT: Neoadjuvant chemotherapy
NPV: Negative predictive value
OC: Ovarian cancer
PDS: Primary debulking surgery
PERCIST: PET Response Criteria in Solid Tumors
PET/CT: Positron emission tomography/computed tomography
PPV: Positive predictive value
Sen: Sensitivity
Spe: Specificity
SUVmax: Maximum standardised uptake value

## Declarations

### Guarantor

The scientific guarantor of this publication is LEE Elaine Yuen Phin.

### Statistics and biometry

No complex statistical methods were necessary for this paper.

### Study subjects or cohorts overlap

Parts of the cohort from this study (42 out of the 46 patients from the prospective cohort) were included in the Proceedings accepted by the 22nd International Cancer Imaging Society Meeting and Annual Teaching Course in London.

### Methodology

• prospective

• diagnostic or prognostic study

• multicentre study
